# Diagnostic discordance and differential diagnoses of community-acquired pneumonia in the emergency department

**DOI:** 10.1186/s12873-026-01641-5

**Published:** 2026-06-12

**Authors:** Susanne Simon, Jan Fuge, Christopher Alexander Hinze, Santiago Ewig, Tobias Welte, Torben Brod, Jessica Rademacher

**Affiliations:** 1 Department of Respiratory Medicine and Infectious Diseases, Medical School Hannover, Carl-Neuberg-Strasse 1, 30625 Hannover, Germany; 2 Biomedical Research in Endstage and Obstructive Lung Disease (BREATH), German Center for Lung Disease (DZL), Medical School Hannover, Hannover, Germany; 3 Department of Respiratory and Infectious Diseases, Thoraxzentrum Ruhrgebiet, EVK Herne and Augusta-Kranken-Anstalt Bochum, Bochum, Germany; 4 Department of Emergency Medicine, Medical School Hannover, Hannover, Germany

**Keywords:** Community-acquired pneumonia, CAP, Emergency department, Diagnostic discordance, Differential diagnosis, Misdiagnosis

## Abstract

**Background:**

Community-acquired pneumonia (CAP) is a common reason for presentation to the emergency department (ED), but accurate diagnosis is challenging due to overlapping and often nonspecific clinical features. Data on the frequency and spectrum of alternative diagnoses in patients initially diagnosed with CAP in the ED are limited. The aim of this study was to evaluate the diagnostic concordance of an initial ED diagnosis of CAP, characterize common alternative discharge diagnoses, and analyze clinical, laboratory, imaging utilization, and vital sign parameters associated with confirmed CAP.

**Methods:**

We conducted a retrospective analysis of diagnostic concordance and differential diagnoses in 1,385 adult patients who presented to the ED of Hannover Medical School and were initially diagnosed with CAP. Initial ED diagnoses were compared with discharge diagnoses.

**Results:**

The cohort comprised 37% female and 63% male patients with a median age of 71 years (IQR 58–79). Comparison between initial ED and discharge diagnoses demonstrated that suspected CAP was confirmed in 52% (*n* = 663) of cases. The most common differential diagnoses were other pulmonary conditions (*n* = 297, 41%), cardiac conditions (*n* = 150, 21%), and renal conditions (*n* = 95, 13%). Patients with confirmed CAP showed significantly higher C-reactive protein (CRP) levels (82 mg/L [IQR 37–159] vs. 57 mg/L [IQR 22–127]; *p* < 0.001) and slightly higher body temperature (37.1 °C [IQR 36.4–38.0] vs. 36.8 °C [IQR 36.2–37.7]; *p* < 0.001) than those with alternative diagnoses.

**Conclusion:**

Only about half of patients initially diagnosed with CAP in the emergency setting had a confirmed diagnosis at discharge, indicating that CAP in this context should be regarded as a working diagnosis rather than a definitive entity. Isolated laboratory, imaging, and vital sign parameters showed limited ability to distinguish CAP from alternative diagnoses, highlighting the importance of structured reassessment after admission to confirm or revise the initial diagnosis and to reduce misclassification and unnecessary antibiotic use.

**Supplementary Information:**

The online version contains supplementary material available at 10.1186/s12873-026-01641-5.

## Background

Community-acquired pneumonia (CAP) is one of the most common infectious diseases worldwide and a frequent reason for presentation to the emergency department (ED). CAP is associated with substantial morbidity and mortality, particularly in elderly and multimorbid patients [1–3]. In Germany, the incidence of CAP is estimated at approximately 300–400 cases per 100,000 inhabitants annually [4], while global incidence rates are estimated at around 10–12 cases per 1,000 adults [2].

In the ED, diagnoses are typically established as initial working diagnoses based on clinical presentation, laboratory findings, and imaging, often under conditions of time pressure and diagnostic uncertainty. Previous studies have reported considerable discordance between admission and discharge diagnoses, with concordance rates ranging from as low as 24% to over 80% depending on the setting [5, 6]. Diagnostic uncertainty is particularly pronounced for infectious diseases. In older patients, provisional ED diagnoses of infections frequently differ from final inpatient diagnoses, with relevant rates of both missed infections and overdiagnosis, especially for pulmonary infections [7, 8]. In this context, CAP represents one of the most frequently overdiagnosed infectious conditions in the ED, with studies showing that up to 30% of initially suspected cases are not confirmed during hospitalization [5, 7, 8].

Differentiating CAP from alternative causes of respiratory symptoms, such as heart failure, pulmonary embolism (PE), interstitial lung disease (ILD), or infectious and non-infectious inflammatory conditions, is essential to ensure appropriate patient management, particularly in the ED setting, where early diagnostic decisions guide initial treatment strategies. Misclassification may result in delayed diagnosis of the true underlying disease, unnecessary antibiotic exposure, inappropriate hospital admissions, and increased healthcare resource utilization. Recent studies have further highlighted that diagnostic misclassification may also lead to short-term adverse events for individual patients and undermine antimicrobial stewardship efforts [7, 9]. In the context of the growing global burden of antimicrobial resistance, avoiding unnecessary anti-infective therapy represents an increasingly important clinical objective [10]. However, there is a lack of real-world data specifically characterizing the spectrum and frequency of alternative diagnoses in patients presenting to the emergency department with suspected CAP.

The aim of this study therefore was to (1) quantify diagnostic concordance between the initial ED diagnosis of CAP and the final hospital diagnosis, (2) systematically characterize the spectrum and frequency of alternative diagnoses in patients with suspected CAP, and (3) analyze routinely available clinical, laboratory, imaging, and vital sign parameters associated with confirmed CAP.

## Methods

**Fig. 1:**
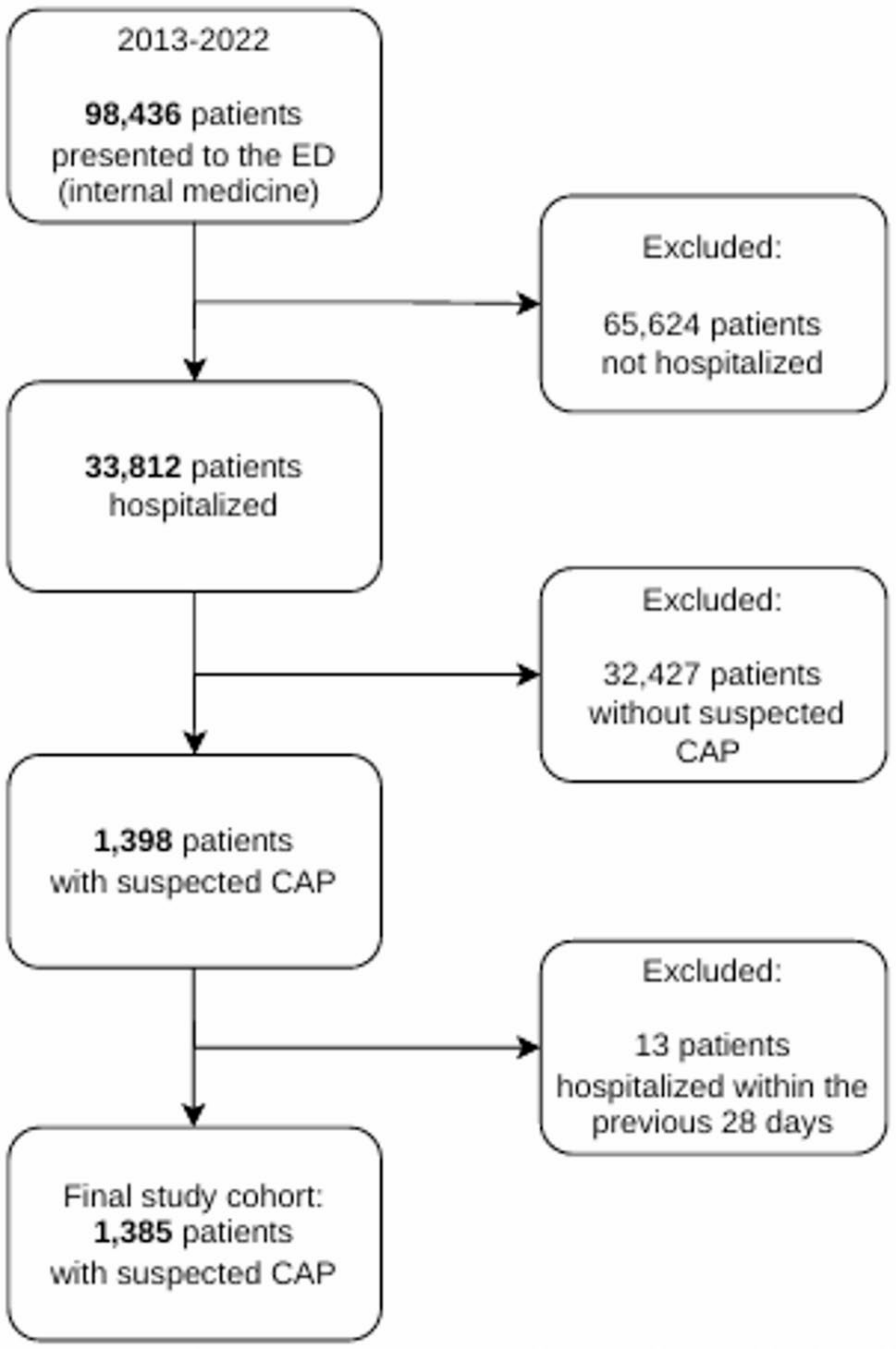
Flowchart of patient selection and study cohort formation for patients presenting to the emergency department with suspected community-acquired pneumonia. CAP = community-acquired pneumonia, ED = Emergency department

We conducted a retrospective analysis of patients presenting to the emergency department of Hannover Medical School, a university hospital and tertiary referral center in northern Germany, between 2013 and 2022, who received an initial diagnosis of community-acquired pneumonia in the ED. During this period, approximately 90,000 patients presented to the ED and were primarily assessed by a resident in internal medicine or one of its subspecialties (postgraduate year 1–6; six-year curriculum in Germany). All clinical data were retrospectively extracted from the electronic health records and documentation systems. Patients were eligible for inclusion if an initial diagnosis of CAP had been documented in the electronic health record by the treating resident in the ED. The initial ED diagnosis was based on routine clinical assessment and physician documentation, including symptoms, physical examination, laboratory findings, and chest radiography available at presentation. No additional retrospective standardization of the initial CAP diagnosis was performed. COVID-19-associated CAP cases were not excluded. All patients underwent chest radiography; however, imaging findings were not systematically reviewed by a board-certified radiologist before the initial diagnosis of CAP was made, reflecting real-world ED conditions. To assess the concordance between the initial ED diagnosis and the final in-hospital diagnosis, admission diagnoses were compared with discharge diagnoses as documented in the hospital information system, which were based on a more comprehensive evaluation including clinical, laboratory, and imaging information obtained during hospitalization. Chest radiograph findings were reassessed by senior attending physicians during the clinical course. Given the retrospective design and the structure of the available dataset, no post hoc reclassification according to more recent pneumonia framework concepts, including pneumonia in immunocompromised patients, was performed. Patients were categorized into those whose diagnosis of CAP was confirmed at discharge and those who received alternative final diagnoses. Alternative diagnoses were categorized into predefined groups (pulmonary, cardiac, renal, musculoskeletal, gastrointestinal, and miscellaneous) based on the final discharge diagnosis. Key laboratory parameters analyzed included C-reactive protein (CRP) (mg/L), leukocyte count (thsd/µl), creatinine levels (µmol/L), estimated glomerular filtration rate (eGFR) (ml/min) and procalcitonin (PCT) levels (µg/L). In addition, the use of imaging modalities at presentation (chest radiography and chest CT) as well as routinely documented vital signs, including body temperature (°C), heart rate (bpm), respiratory rate (/min), systolic blood pressure (mmHg), and peripheral oxygen saturation (SpO₂), were analyzed. Information on supplemental oxygen use at the time of vital sign assessment was not consistently available in the retrospective dataset. Patient selection and cohort formation are summarized in Fig. 1.

Statistical analyses were performed using R software (version 4.5.2; R Foundation for Statistical Computing, Vienna, Austria) within the RStudio environment. Continuous variables are presented as median and interquartile range (IQR) and were analyzed using the Mann–Whitney U test for comparisons between two groups and the Kruskal-Wallis test for comparisons across multiple groups. Categorical variables were compared using chi-square (χ²) tests. Univariable and multivariable logistic regression analyses were conducted to assess associations between independent variables and the outcome. Data visualization included Sankey diagrams to illustrate flow and relationships between categorical variables, and violin plots to display the distribution and density of continuous variables across groups. A two-sided p-value of < 0.05 was considered statistically significant. The study was approved by the local ethics committee of Hannover Medical School (approval number: (15/12/2022; No. 10670_B0_K_2022). The requirement for informed consent was waived due to the retrospective design.

## Results

**Fig. 2:**
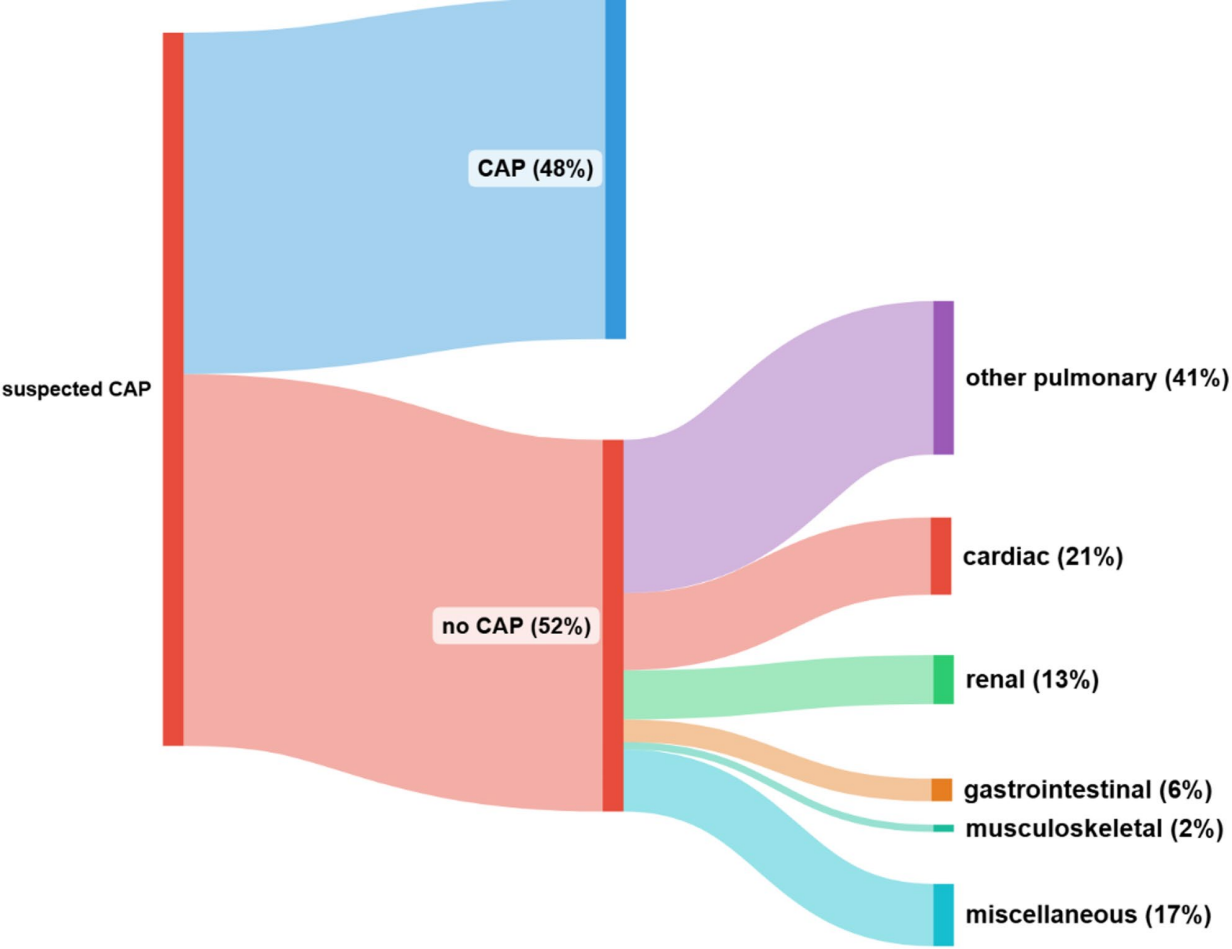
Sankey-chart: subgrouping of all patients admitted with a suspected diagnosis of community-acquired pneumonia (*n* = 1385). CAP = Community-acquired pneumonia

The study cohort consisted of *n* = 1,385 patients, of whom 37% were female (*n* = 508) and 63% were male (*n* = 877), with a median age of 71 years (IQR 58–79) (Table 1). Of all cases diagnosed with CAP in the ED, 52% (*n* = 663) were confirmed upon discharge. The remaining 48% (*n* = 722) were found to have alternative diagnoses as shown in Fig. 2.

The most common alternative diagnoses included other pulmonary conditions (41%, *n* = 297), cardiac conditions (21%, *n* = 150), renal conditions (13%, *n* = 95), musculoskeletal causes (2%, *n* = 14), gastrointestinal conditions (6%, *n* = 44), and miscellaneous causes (17%, *n* = 122). Other pulmonary conditions included lower respiratory tract infections (LRTI) (*n* = 85), infection of the upper respiratory tract (*n* = 9), pleural effusion or empyema (*n* = 17), newly diagnosed or progress of bronchial carcinoma (*n* = 26), exacerbation of an underlying pulmonary disease such as cystic fibrosis, chronic obstructive pulmonary disease (COPD), asthma, ILD, bronchiectasis, chronic lung allograft dysfunction (*n* = 78), PE (*n* = 25) with or without congestive pneumonia (*n* = 19), lung metastases (*n* = 1), pneumothorax (*n* = 2) and pleurisy (*n* = 3) or rare conditions such as cryptogenic organizing pneumonia or radiation pneumonitis (*n* = 33).

Patients with confirmed CAP had higher CRP levels than patients without confirmed CAP (82 mg/L [IQR 37–159] vs. 57 mg/L [IQR 22–127]; *p* < 0.001) as shown in Table 1. Apart from CRP, no significant differences between patients with confirmed CAP and those with alternative diagnoses regarding age, leukocyte count, renal function parameters, or PCT levels were observed (Table 1). In the Kruskal-Wallis analysis across diagnostic categories, significant differences were observed for age, CRP, creatinine, and eGFR (all *p* < 0.001), whereas leukocyte count and procalcitonin did not differ significantly between groups.

**Table 1:** Patient characteristics and laboratory parameters at initial presentation to the emergency department in patients with confirmed CAP versus no CAP (Mann-Whitney U test, Chi² *), and the various differential diagnoses compared with each other using the Kruskal-Wallis test (#), presented as median with IQR

	All	Community- acquired pneumonia (*n* = 663)	No community- acquired pneumonia (*n* = 722)	*p*	Other pulmonary (*n* = 298)	Cardiac (*n* = 150)	Renal (*n* = 95)	Muscolo- skeletal (*n* = 14)	Gastrointestinal (*n* = 44)	Miscellaneous (*n* = 121)	*p*#
Age (years)	*n* = 1385 71 (58–79)	*n* = 663 71 (57–80)	*n* = 722 71 (59–79)	0.579	67 (53–77)	77 (69–84)	76 (64–82)	72 (49–80)	67 (55–76)		
CRP (mg/L)	*n* = 1189 69 (28–142)	*n* = 572 82 (37–159)	*n* = 617 57 (22–127)	**< 0.001**	*n* = 262 51 (22–121)	*n* = 127 51 (17–97)	*n* = 86 65 (31–157)	*n* = 10 41 (9-168)	*n* = 35 44 (17–130)	*n* = 97 87 (32–167)	< 0.001
Leukocytes (thsd/µl)	*n* = 1201 11 (8–15)	*n* = 573 11 (7–15)	*n* = 628 11 (8–15)	0.363	*n* = 262 11 (8–15)	*n* = 131 10 (8–13)	*n* = 86 13 (9–13)	*n* = 10 15 (8–21)	*n* = 36 11 (7–14)	*n* = 103 11 (8–15)	0.333
Creatinine (µmol/L)	*n* = 1199 92 (70–132)	*n* = 575 89 (68–130)	*n* = 624 95 (73–138)	0.008	*n* = 261 84 (68–109)	*n* = 132 112 (91–174)	*n* = 86 127 (90–249)	*n* = 10 73 (50–113)	*n* = 35 101 (75–156)	*n* = 100 87 (66–115)	< 0.001
eGFR (ml/min)	*n* = 1199 60 (41–80)	*n* = 575 60 (42–81)	*n* = 624 60 (39–79)	0.165	*n* = 261 60 (53–86)	*n* = 132 49 (28–60)	*n* = 86 41 (21–61)	*n* = 10 71 (55–112)	*n* = 35 60 (36–74)	*n* = 100 60 (47–95)	< 0.001
PCT (µg/L)	*n* = 628 0.2 (0.1–0.7)	*n* = 320 0.2 (0.1–0.9)	*n* = 308 0.2 (0.1–0.5)	0.007	*n* = 148 0.1 (0.1–0.3)	*n* = 53 0.2 (0.1–0.5)	*n* = 33 0.3 (0.15-1.0)	*n* = 3 0.1 (0.1- -)	*n* = 11 0.7 (0.1–2.3)	*n* = 60 0.1 (0.1–0.6)	0.063
Sex				0.435*							
Female – n (%)	*n* = 508	236 (36%)	272 (37%)								
Male – n (%)	*n* = 877	427 (64%)	450 (63%)								

CAP = community-acquired pneumonia, CRP = C-reactive protein, eGFR = estimated Glomerular Filtration Rate, IQR = interquartile range, PCT = procalcitonin

Analyses of imaging modalities and vital signs at initial presentation to the emergency department are shown in Table 2. Imaging data were available for 648 patients with confirmed CAP and 710 patients without confirmed CAP. Chest X-ray was performed in the majority of patients. Two-view chest radiographs represented the most frequently used imaging modality (58% vs. 53%, *p* = 0.118), whereas chest CT was performed more frequently, but not significantly, in patients without confirmed CAP (13% vs. 11%, *p* = 0.102). Combined chest CT and one-view chest radiography was more common among patients without confirmed CAP (5% vs. 3%, *p* = 0.015). Patients with confirmed CAP showed a higher body temperature (37.1 °C [36.4–38.0] vs. 36.8 °C [36.2–37.7], *p* < 0.001) and more frequently presented with temperatures > 38 °C (25.0% vs. 18.2%, *p* = 0.004), whereas temperatures ≤ 36 °C did not differ significantly between groups (16.4% vs. 17.7%, *p* = 0.577). Heart rate values were marginally higher (94 bpm [IQR 80–109] vs. 90 bpm [IQR 78–104], *p* = 0.002) at presentation. Systolic blood pressure, respiratory rate, and SpO₂ were largely comparable between groups.

**Table 2:** Imaging modalities and vital signs at initial presentation to the emergency department in patients with confirmed CAP vs. no CAP (Mann-Whitney U test, Chi² *), presented as n (%) or median with IQR. Percentages for imaging modalities are based on patients with available imaging data in the respective group

Imaging modality	Community-acquired pneumonia (*n* = 648)	No community- acquired pneumonia (*n* = 710)	*p*
Chest X-ray one view n (%)	260 (40)	320 (45)	0.066*
Chest X-ray two views n (%)	376 (58)	382 (53)	0.118*
Chest CT n (%)	68 (10)	95 (13)	0.102*
Chest CT + chest X-ray one view n (%)	17 (3)	37 (5)	0.015*
Chest CT + chest X-ray two views n (%)	29 (5)	33 (4)	0.879*
Vital signs (median, IQR)			p
Temperature (°C)	*n* = 628 37.1 (36.4–38.0)	*n* = 660 36.8 (36.2–37.7)	< 0.001
Heart rate (bpm)	*n* = 645 94 (80–109)	*n* = 693 90 (78–104)	0.002
Blood pressure systolic (mmHg)	*n* = 631 131 (116-147.5)	*n* = 679 133 (118–150)	0.183
Respiratory rate (/min)	*n* = 565 17 (16–20)	*n* = 594 17 (16–20)	0.984
SpO₂ (%)	*n* = 631 95 (92–97)	*n* = 680 95 (92–97)	0.050

bpm = beats per minute, CAP = community-acquired pneumonia, CRP = C-reactive protein, CT = computed tomography, IQR = interquartile range, SpO₂ = peripheral oxygen saturation, X-ray = radiograph

Supplementary analyses of imaging modalities and routinely documented vital signs across diagnostic categories are shown in Supplementary Table S1.

In univariate logistic regression analyses, higher CRP levels and body temperature were associated with confirmed CAP, whereas leukocyte count, age, procalcitonin, heart rate, SpO₂, and respiratory rate were not significant predictors. In the multivariable model, both higher CRP levels (OR 1.03 per 10 mg/L increase, 95% CI 1.02–1.05; *p* < 0.001) and higher body temperature (OR 1.23, 95% CI 1.10–1.37; *p* < 0.001) remained independently associated with confirmed CAP. Overall, the model showed limited explanatory power (Nagelkerke R² = 0.050) (Table 3).

**Table 3:** Univariate and multivariable logistic regression analysis of routinely documented laboratory and vital parameters at initial presentation to the emergency department associated with confirmed community-acquired pneumonia. Continuous variables are shown per specified unit increase

	Univariate OR (95% CI)	*p*-value	Multivariable OR (95% CI)	*p*-value
CRP (per 10 mg/L)	1.03 (1.02–1.04)	< 0.001	1.03 (1.02–1.05)	< 0.001
Leukocytes (thsd/µl)	1.00 (0.99–1.02)	0.707	-	-
Age (per 10 years)	0.97 (0.91–1.03)	0.347	-	-
Procalcitonin (µg/L)	1.00 (0.98–1.02)	0.914	-	-
Temperature (°C)	1.24 (1.12–1.36)	< 0.001	1.23 (1.10–1.37)	< 0.001
Heart rate (bpm)	1.00 (1.00-1.01)	0.161	-	-
SpO₂ (%)	0.98 (0.96-1.00)	0.124	-	-
Respiratory rate (/min)	1.00 (0.97–1.03)	0.899	-	-

Multivariable model performance: Nagelkerke R² = 0.050, CRP = C-reactive protein, eGFR = estimated Glomerular Filtration Rate, SpO₂ = peripheral oxygen saturation

## Discussion

This study highlights the significant diagnostic challenge of CAP in the emergency department setting, with only half of the initial diagnoses being confirmed at hospital discharge. These findings suggest that CAP in the emergency setting often represents a clinical working diagnosis, encompassing a broad spectrum of pulmonary and extrapulmonary conditions with overlapping symptoms. CRP levels were associated with a confirmed CAP diagnosis and may provide support in the diagnostic assessment.

Our results align with recent studies that have reported diagnostic inaccuracies. Gupta et al. highlighted that a substantial proportion of hospitalized adults were misdiagnosed with CAP, largely due to non-specific symptoms and overlapping clinical features with other diseases [7, 11]. The most common symptoms of CAP, including cough, dyspnea, and sputum production, are highly nonspecific, and no single clinical sign reliably establishes the diagnosis [12]. Even non-respiratory infections such as urinary tract infections may present with respiratory symptoms [13]. In our cohort, this diagnostic uncertainty was reflected by the broad spectrum of alternative diagnoses, including mostly pulmonary conditions, as well as cardiac, renal, musculoskeletal (e.g., inspiratory chest pain and cough due to rib fracture) and gastrointestinal diagnoses (e.g., cholangitis with radiating pain to the chest or cough associated with gastroesophageal reflux disease).

Our observed rate of diagnostic discordance of nearly 50% is higher than the approximately 12% reported in recent multicenter cohorts [7, 14] and also exceeds rates of around 30% described in previous single-center studies [5, 8]. Two main factors may explain this difference. First, we applied a relatively strict definition of incorrect CAP diagnosis based solely on the discharge diagnosis, which may have increased the apparent misclassification rate. This approach is consistent with the methodology used by Atamna et al. [5]. However, their analysis was limited to infectious diagnoses, whereas our study also included a broad spectrum of non-infectious differential diagnoses. As discharge diagnoses were used as the reference standard in this retrospective analysis, some degree of overestimation of diagnostic discordance cannot be excluded. Second, our study was conducted at a large tertiary care emergency department and transplant center, including a heterogeneous patient population with a relevant proportion of immunocompromised patients. In this population, typical clinical and radiographic findings may be less pronounced or atypical [7, 15–17]. Immunocompromised patients represent a distinct and heterogeneous population that is not adequately covered by standard CAP definitions or guidelines, and for whom no universally accepted diagnostic framework exists, further complicating the clinical assessment of CAP in routine practice [18, 19].

The diagnostic discrepancies observed in our study have important clinical implications. Misclassification may result in delayed diagnosis of the true underlying disease and may contribute to inappropriate resource utilization and hospital admissions, in addition to unnecessary antibiotic use, which increases the risk of adverse effects and contributes to antibiotic resistance [7, 10]. However, the overall rate of diagnostic discordance should not be equated with the rate of clinically relevant overtreatment. Some patients categorized as not having CAP still had infectious or inflammatory conditions requiring antibiotic therapy, such as empyema, exacerbated interstitial lung disease, or abdominal sepsis. Depending on the definition applied, the proportion of patients who truly received unnecessary antibiotics may therefore be substantially lower than the overall rate of diagnostic discordance.

In our study, CRP levels were significantly higher in patients with confirmed CAP compared to those with alternative diagnoses, while other biomarkers such as leukocyte count showed no significant association and PCT demonstrated only limited discriminatory value. The question of which biomarker is most suitable in this setting remains controversial. A systematic review reported only limited predictive value of CRP for the diagnosis of CAP [20], while a more recent meta-analysis found CRP to be superior to PCT for predicting CAP in the outpatient setting [21]. Other studies suggest that CRP may provide modest additional diagnostic certainty at thresholds above 30 mg/L when clinical assessment remains inconclusive, whereas PCT does not appear to add clinically relevant information in primary care [22].

Similarly, routinely documented vital signs showed only limited discriminatory value in our cohort. Apart from slightly higher body temperature and heart rate in patients with confirmed CAP, respiratory rate, systolic blood pressure, and SpO₂ were largely comparable between groups. This is consistent with previous studies showing that isolated vital sign abnormalities have limited diagnostic accuracy for CAP in the ED setting, although the absence of abnormal vital signs may lower the probability of CAP [12, 23]. In addition, respiratory rate is frequently estimated rather than systematically measured in routine clinical care [24, 25], and interpretation of SpO₂ values is limited by inconsistent documentation of supplemental oxygen administration [25]. Recent data further suggest that the overall clinical impression may be more informative than isolated vital sign abnormalities when evaluating patients with suspected CAP [23].

Despite the independent association between CRP levels and body temperature with confirmed CAP in multivariable analyses, the overall explanatory power of the regression model remained limited, suggesting that isolated laboratory and vital sign parameters are insufficient to reliably distinguish CAP from alternative diagnoses in the ED.

To improve diagnostic accuracy, different strategies may be considered. In our cohort, chest radiography represented the most frequently used imaging modality. Chest radiography has limited sensitivity and specificity for CAP diagnosis, particularly in complex patient populations [26]. A more systematic approach to imaging interpretation may help to reduce diagnostic uncertainty. Several studies have demonstrated that chest CT can lead to reclassification of the diagnosis in a substantial proportion of patients, often resulting in a reduction of CAP diagnoses and identification of alternative conditions [27–29]. However, only a minority of patients in our cohort underwent CT imaging, and CT was not performed substantially more frequently in patients with subsequently confirmed CAP. Therefore, our data do not allow conclusions regarding the potential impact of CT imaging on diagnostic accuracy in this setting. Future approaches, including artificial intelligence-assisted imaging interpretation, may further support diagnostic assessment [30, 31]. Second, adopting standardized diagnostic approaches may help reduce variability and improve consistency in CAP diagnoses. Although our retrospective study was not designed to derive a formal diagnostic algorithm, the findings underline the frequent need for diagnostic reassessment during hospitalization [12]. Standardized assessment may integrate clinical condition, inflammatory markers, imaging findings, and, where appropriate, microbiological sampling at initial presentation [12, 32, 33], while common alternative diagnoses, particularly other pulmonary, cardiac, and renal conditions, should be actively considered. After admission, reassessment within 48–72 h may be useful and can include review of microbiological and radiological findings and reevaluation of ongoing antimicrobial therapy, which is also addressed in current CAP guidelines [32, 33].

This study has several limitations. Its single-center design and reliance on admission and discharge diagnoses without verification against standardized diagnostic criteria may limit generalizability. In addition, reliance on documented diagnoses may be subject to documentation and coding bias. Due to the retrospective design it remains unclear at which point during hospitalization the diagnosis was revised. Also, we did not systematically differentiate between immunocompromised and non-immunocompromised patients, and no post hoc reclassification according to more recent pneumonia framework concepts was performed. While this reflects the pragmatic approach in the emergency setting, a more detailed analysis of specific patient subgroups would be of clinical interest and should be addressed in future studies. Furthermore, we did not assess antibiotic use or clinical outcomes. An evaluation of severity scores is missing, although all included patients were considered sufficiently ill to require hospital admission. The long study period from 2013 to 2022 may have introduced temporal heterogeneity due to evolving diagnostic practices and the impact of the COVID-19 pandemic. However, only a small proportion of patients were ultimately diagnosed with COVID-19 pneumonia (*n* = 21, 1.5%).

In conclusion, the high rate of diagnostic discordance observed in our study illustrates the inherent uncertainty of CAP diagnosis at the time of emergency department presentation, where decisions must often be made rapidly on the basis of limited and nonspecific clinical information. In this context, the initial diagnosis of CAP should be regarded as a pragmatic working diagnosis rather than a definitive entity. Our findings demonstrate that patients with suspected CAP may have a wide spectrum of pulmonary and extrapulmonary differential diagnoses, and that a correct diagnosis is not always achievable at first assessment. Therefore, systematic reassessment after admission may represent an important component of the diagnostic process in patients with suspected CAP, allowing confirmation or revision of the initial diagnosis once additional clinical, laboratory, and imaging data become available. By emphasizing this iterative diagnostic approach, clinicians may facilitate more appropriate diagnostic and antimicrobial decision-making during hospitalization.

## Supplementary Information

Below is the link to the electronic supplementary material.

Supplementary Material 1

## Data Availability

The datasets used and/or analysed during the current study are available from the corresponding author on reasonable request.

## Abbreviations

CAP: Community-acquired pneumonia
COPD: Chronic obstructive pulmonary disease
CRP: C-reactive protein
ED: Emergency Department
eGFR: Estimated Glomerular Filtration Rate
ILD: Interstitial lung disease
LRTI: Lower respiratory tract infections
PCT: Procalcitonin
PE: Pulmonary embolism
SpO₂: Peripheral oxygen saturation
X-ray: Chest radiograph
