# Supplementary material for: Diagnostic discordance and differential diagnoses of community-acquired pneumonia in the emergency department
Source: BMC Emerg Med. 2026 Jun 12;26:171. doi: 10.1186/s12873-026-01641-5 (PMC13263920; doi:10.1186/s12873-026-01641-5)
Supplement: Supplementary file 1 — Supplementary Material 1 [file 12873_2026_1641_MOESM1_ESM.docx]

Supplemental material

**Table S1.** Imaging modalities and vital signs across different alternative diagnoses in patients initially admitted with suspected community-acquired pneumonia, presented as n (%) or median with interquartile range (IQR). Group comparisons were performed using chi-square (χ²) (*) analysis for categorical variables and the Kruskal-Wallis test (#) for continuous variables. Percentages refer to the respective diagnostic subgroup. Some patients underwent more than one imaging modality; therefore, percentages may exceed 100%.

| Imaging modality | Other pulmonary (n = 291) | Cardiac  (n = 149) | Renal  (n = 94) | Muscolo-  skeletal  (n = 14) | Gastrointestinal  (n = 43) | Miscellaneous  (n = 119) | p* |
| --- | --- | --- | --- | --- | --- | --- | --- |
| *Chest X-ray*  *one view n (%)* | 78 (27) | 82 (55) | 62 (66) | 8 (57) | 27 (63) | 63 (53) | <0.001 |
| *Chest X-ray*  *two views n (%)* | 198 (68) | 71 (48) | 34 (36) | 6 (43) | 15 (35) | 58 (49) | <0.001 |
| *Chest CT n (%)* | 68 (23) | 61 (41) | 10 (11) | 1 (7) | 2 (5) | 7 (6) | <0.001 |
| *Chest CT + Chest X-ray one view n (%)* | 17 (6) | 17 (11) | 7 (7) | 1 (7) | 2 (5) | 5 (4) | 0.005 |
| *Chest CT + Chest X-ray two views n (%)* | 25 (9) | 2 (1) | 0 (0) | 0 (0) | 1 (2) | 5 (4) | 0.003 |
| Vital signs (median, IQR) |  |  |  |  |  |  | p# |
| *Temperature (°C)* | n = 274  36.8  (36.2-37.5) | n = 141  36.6  (36.1-37.1) | n = 90  37.1  (36.4-38.2) | n = 11  36.5  (36.2-37.5) | n = 39  36.6  (36.2-37.5) | n = 105  37.0  (36.4-38.0) | <0.001 |
| *Heart rate (bpm)* | n = 290  92 (80-104) | n = 148  87 (70-100) | n = 92  88 (76-105) | n = 13  87 (86-99) | n = 40  88 (80-104) | n = 110  93 (83-104) | 0.142 |
| *Blood pressure systolic (mmHg)* | n = 282  133  (119-148) | n = 148  139 (118-165) | n = 86  129 (116-147) | n = 13  149 (130-153) | n = 40  128 (112-137) | n = 110  132 (114-148) | <0.001 |
| *Respiratory rate (/min)* | n = 239  17 (16-20) | n = 137  18 (16-20) | n = 79  17 (16-18) | n = 11  18 (16-22) | n = 34  18 (16-20) | n =94  18 (16-20) | 0.496 |
| *SpO*₂ *(%)* | n = 281  95 (92-97) | n = 149  95 (92-96) | n = 91  95 (93-96) | n = 13  97 (95-98) | n = 40  96 (93-97) | n = 106  95 (93-98) | 0.251 |

bpm = beats per minute, CT = computed tomography, IQR = interquartile range, SpO₂ = peripheral oxygen saturation
